# Air Embolism After Central Venous Catheter Insertion via the Internal Jugular Vein: A Case Report

**DOI:** 10.7759/cureus.87912

**Published:** 2025-07-14

**Authors:** Ibrahim Saleh, Ayman Emara, Zeyad Khalil

**Affiliations:** 1 Department of Surgery, Montefiore Medical Center, Albert Einstein College of Medicine, Bronx, USA; 2 Department of Critical Care Medicine, Alexandria University Faculty of Medicine, Alexandria, EGY; 3 College of Medicine, 6th of October University, Cairo, EGY

**Keywords:** air embolism, case report, central venous catheterization, internal jugular vein catheterization, refractory septic shock

## Abstract

We present the case of a 50-year-old female admitted to the intensive care unit with altered mental status and cardiovascular compromise. Despite vasopressor support via a central venous catheter in the right internal jugular vein, she remained in shock. Chest CT revealed an iatrogenic air embolism involving the right ventricle. The embolism resolved after catheter removal, Trendelenburg and left lateral decubitus positioning, and mechanical ventilation. However, the patient subsequently developed refractory septic shock, unresponsive to broad-spectrum antibiotics and maximum norepinephrine support, leading to her death on day six. This case highlights the importance of early detection and prompt management of air embolism.

## Introduction

Central venous catheterization (CVC) is a widely performed procedure, particularly in emergency settings for fluid and inotropic administration. The main indications for CVC insertion include the administration of high-dose vasopressors and the lack of available peripheral access. Several access sites are used for CVC insertion, including the internal jugular vein (IJV), subclavian vein, and femoral vein [1]. The introduction of ultrasound (US) and fluoroscopic guidance has significantly improved the safety and precision of CVC placement compared to traditional anatomical landmark-based techniques [2]. A meta-analysis of 10 studies involving 2,168 patients (six real-time US, one static US, and three Doppler US studies) showed that US guidance significantly reduced overall complications (odds ratio, 95% CI: 0.53, 0.41-0.69) compared with the landmark guidance [3]. Despite these advancements, complications still occur during CVC placement. These complications include arterial puncture, venous thrombosis, pneumothorax, hemothorax, arrhythmias, and air embolism [1]. 

Air embolism is one of the most critical and potentially fatal complications of CVC insertion. The incidence ranges from 0.03% to 2%, with a mortality rate reaching up to 50% in severe cases. The rapid entry of air into the heart or pulmonary circulation can result in cardiovascular collapse and multi-organ failure if not promptly recognized and treated [4]. Early detection and timely intervention are crucial to improving patient outcomes in such cases. The severity of symptoms depends on the volume and rate of air entry, as well as its destination within the vascular system. Air embolism can obstruct the right ventricular outflow tract or pulmonary arteries, leading to impaired venous return, reduced cardiac output, and cardiovascular collapse [5]. Animal studies have reported lethal air volumes of 0.5-0.75 mL/kg in rabbits and 7.5-15.0 mL/kg in dogs, whereas in humans, the lethal air volume is 300-500 mL when introduced rapidly [6].

The IJV is commonly chosen for CVC placement due to its compressibility, straight anatomical path to the superior vena cava, and lower risk of pneumothorax compared to the subclavian approach. Subclavian access is more prone to air embolism and pneumothorax, while femoral access carries a greater risk of infection and thrombosis. The IJV approach offers a balanced risk profile but is highly dependent on operator expertise [3]. Factors such as lack of US guidance, limited experience, and emergency conditions can increase the chance of mechanical errors and air embolism. Air embolism generally occurs when a pressure gradient enables air to enter the venous system, particularly when central venous pressure is low, such as in hypovolemia, during deep inspiration, and when the catheter or insertion site is exposed to ambient air. This risk is amplified by improper technique, inadequate occlusion of the catheter hub, or placing the patient in an upright position [7].

## Case presentation

**Figure 1:**
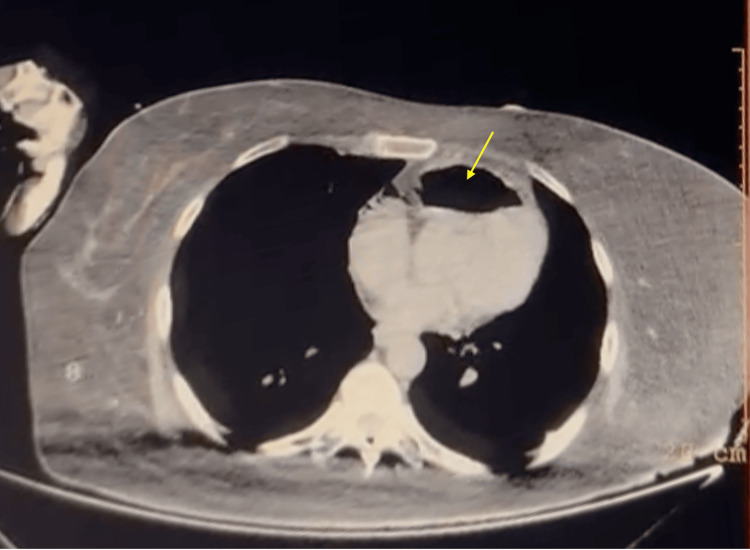
Non-contrast CT of the chest (axial view) showing a distinct radiolucent area (yellow arrow) within the right ventricle, consistent with the presence of air embolism.

A 50-year-old woman presented to the emergency room in an unconscious shock state. Her vital signs revealed a systolic blood pressure (SBP) of 40 mmHg, a heart rate (HR) of 110 bpm, a respiratory rate of 28 breaths per minute, and a temperature of 98.6 °F (37 °C). On physical examination, she was lethargic and disoriented, but without focal neurological deficits. Chest auscultation revealed normal breath and heart sounds. Her past medical history was unremarkable except for a five-day history of urinary tract infection (UTI). An arterial blood gas (ABG) analyzer (GEM premier 3000, Erba, India) on room air showed a pH of 7.28 (7.35-7.45), PaCO₂ of 44 mmHg (35-45), PaO₂ of 98 mmHg (80-105), HCO₃ of 21 mEq/L (22-27), and lactate of 3.2 mmol/L (0-2). She was intubated in a persistent shock state, and a CVC was inserted in the right IJV using landmark guidance. Her neurological status remained unchanged, and she was then admitted to the intensive care unit (ICU) for continued monitoring. Initial management included intravenous vasopressors, with norepinephrine starting at 1 mcg/kg/min. An axial CT scan (Philips [ingenuity], Philips Healthcare, Amsterdam, The Netherlands) performed 30 minutes after CVC insertion showed air in the right ventricle, as shown in Figure 1. The CVC was removed, and the patient was placed on mechanical ventilation in assisted mode and positioned in the Trendelenburg and left lateral decubitus positions (Durant’s maneuver) for 24 hours.

**Figure 2:**
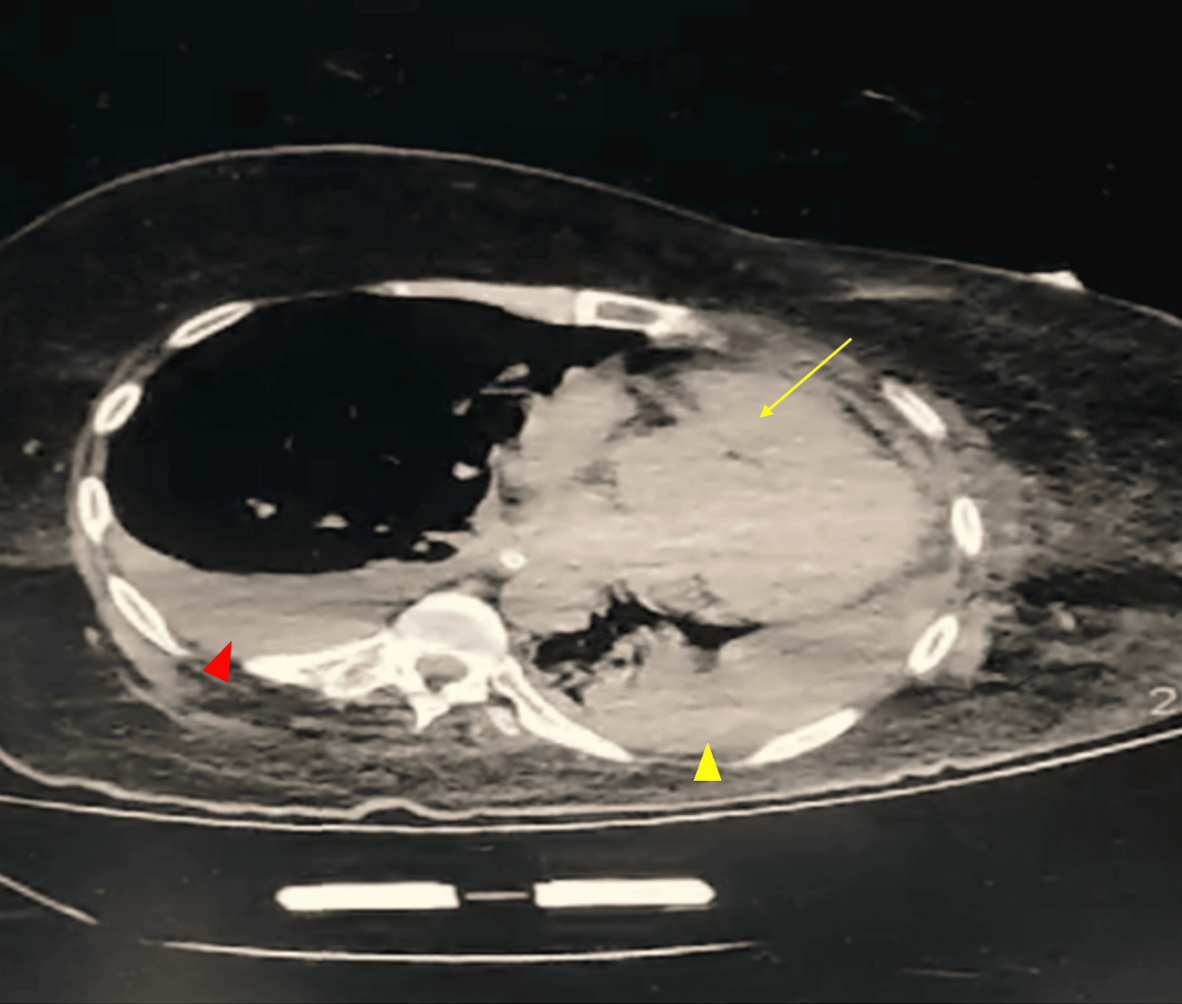
Non-contrast chest CT (axial view) performed 48 hours after admission, showing consolidative patches with air bronchograms in the left lung, relative left lung collapse with mediastinal shift to the left, bilateral basal atelectatic bands, and resolved air embolism (yellow arrow). Additionally, bilateral pleural effusions are present: mild on the right (red arrowhead) and moderate, encysted on the left (yellow arrowhead).

Following catheter removal, the air embolism resolved; however, a follow-up chest CT revealed bilateral basal atelectatic bands, along with dense ground-glass opacity in the lower right pleura, as shown in Figure 2. A screening echocardiogram (Mindray MT3, Shenzhen, China) was then performed, which revealed no evidence of right or left ventricular failure. Another CVC was inserted into the left IJV using the dynamic US insertion guidance without complications. The patient’s hypoxic index (PaO₂/FiO₂) was 245, and her blood pressure was 86/40 mmHg, necessitating further inotropic/vasopressor support (norepinephrine 1 mcg/kg/minute). Samples from sputum, urine, and blood were collected for culture. No growth was seen in the mini-BAL and blood cultures, while the urine culture demonstrated significant growth of Escherichia coli (>100,000 CFU/mL). Laboratory findings were mostly unremarkable except for a leukocyte count of 14,000/μL (4,000-10,000), with 88% neutrophils (40%-75%).

Despite initial stabilization efforts, the patient’s condition continued to deteriorate, marked by worsening hypoxemia and hemodynamic instability. Over the subsequent 48 hours, she remained hemodynamically unstable despite ongoing vasopressor infusion. The patient died within six days of admission due to persistent septic shock, despite broad-spectrum antibiotic therapy (Meropenem and Moxifloxacin) and maximum doses of norepinephrine and hydrocortisone. Table 1 shows the clinical course, chronological timeline, and treatment summary for this case. 

**Table 1:** The clinical course, timeline and treatment summary. *Durant’s maneuver: Used to trap air in the right ventricle apex, preventing obstruction of pulmonary blood flow and reducing hemodynamic instability in venous air embolism. ER, emergency room; SBP, systolic blood pressure; HR, heart rate; RR, respiratory rate; Temp, temperature; ABG, arterial blood gas; pH, potential of hydrogen; PaCO₂, partial pressure of carbon dioxide in arterial blood; PaO₂, partial pressure of oxygen in arterial blood; CVC, central venous catheter; IJV, internal jugular vein; ICU, intensive care unit; BP, blood pressure; CT, computed tomography; SVC, superior vena cava; RA, right atrium; RV, right ventricle; ECHO, echocardiogram; FiO₂, fraction of inspired oxygen; WBC, white blood cell count

Day/time	Clinical events	Vital signs and labs	Imaging/diagnostics	Interventions/treatments
Day 0 (ER Arrival)	Unconscious, in shock state	SBP: 40, HR: 110, RR: 28, Temp: 37°C	ABG: pH 7.28, PaCO₂ 44, PaO₂ 98, Lactate 3.2	Intubation, CVC insertion (right IJV), initiation of norepinephrine (1 mcg/kg/minute)
Day 0 (ICU Admission)	Persistent hypotension, shock state	BP: 86/40 after vasopressors	- CT neck/thorax: Air in SVC, RA, RV, pulmonary artery (iatrogenic air embolism)	- Trendelenburg + left lateral decubitus positioning (*Durant’s maneuver), CVC removal, continued mechanical ventilation - Cultures from sputum, urine, and blood - Initiation of broad-spectrum antibiotics (Meropenem, Moxifloxacin) and Hydrocortisone
Day 1 (Post-Catheter Removal)	Resolution of air embolism, better hemodynamics	PaO₂/FiO₂ ratio: 245	- Follow-up chest CT: Resolved air embolism, Bilateral basal atelectasis, dense ground-glass opacity (Figure 2) - ECHO: no evidence of right or left ventricular failure.	- Reducing norepinephrine dose
Day 2 (ICU Stay)	Hemodynamic instability, signs of sepsis	BP: Persistently low despite vasopressors, WBC: 14,000 (88% neutrophils)	N/A	Continued the same medications (norepinephrine, antibiotics, hydrocortisone)
Days 3-4	Progressive septic shock, refractory hypotension	Worsening hypoxemia, no significant lab improvement	N/A	Escalation to maximum norepinephrine dose, continued antibiotics, and supportive ICU care.
Days 5-6 (Outcome)	Multi-organ failure, refractory septic shock	BP unresponsive to maximum vasopressor support	N/A	No response to therapy, patient deceased due to persistent septic shock

**Figure 3:**
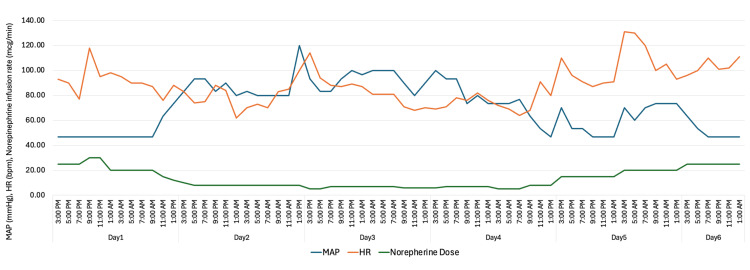
Graphical representation of blood pressure, heart rate, and norepinephrine infusion rate over time.

Figure 3 shows a graphical representation of mean arterial pressure (MAP), HR, and norepinephrine infusion rate over time for this case. Initially, the patient's MAP began to rise, HR stabilized, vasopressor requirements decreased, and FiO₂ demand was reduced. However, by the fourth day, the patient experienced clinical deterioration marked by recurrent tachycardia, hypotension, escalation in vasopressor support, and progression to refractory septic shock.

## Discussion

Central line insertion is a vital procedure frequently performed in emergency rooms and critical care units. It serves several purposes, including fluid resuscitation when peripheral access is not feasible, administration of vasoactive medications, hemodynamic monitoring, infusion of hyperosmolar solutions, and delivery of total parenteral nutrition [5]. Central line insertion carries risks ranging from mild to life-threatening complications, including vascular injuries (arterial or venous), hemothorax, cardiac tamponade, infections, pneumothorax, arrhythmias, and air embolism [1].

In this case, the patient developed a venous air embolism (VAE), a rare but potentially fatal complication. VAE can range from asymptomatic cases to life-threatening presentations, including dyspnea, chest pain, dizziness, acute right-sided heart failure, obstructive shock, or cardiac arrest. Rapid recognition and intervention are key to resolving the air embolism. Most reported cases resolve with immediate catheter removal, Durant’s maneuver, and hyperbaric oxygen therapy when indicated [8,9]. The uniqueness of our case lies in the shock state the patient presented with, which could potentially obscure the early detection and intervention of the air embolism. Despite prompt recognition and management of the air embolism, our patient’s condition deteriorated due to refractory septic shock. The patient remained hemodynamically unstable despite broad-spectrum antibiotics and maximal vasopressor support. Given the severity of her shock, adjunctive therapies such as corticosteroids or early initiation of extracorporeal support could have been considered in similar cases, though evidence remains inconclusive. Corticosteroids, which are recommended in cases of persistent septic shock, were administered to our patient but did not result in improvement. Extracorporeal support was not pursued, as there was no indication for cardiac or respiratory failure warranting such intervention.

Management of VAE includes supportive measures such as high-flow oxygen, mechanical ventilation, intravenous fluids, vasopressors, and positioning in the left lateral decubitus and Trendelenburg positions (Durant’s maneuver) [10]. Definitive therapies include hyperbaric oxygen therapy and manual removal of embolized air [11]. In our case, immediate catheter removal, supportive measures, and Durant’s maneuver improved the patient’s hemodynamics transiently, as evidenced by the follow-up CT findings (Figure 2). While the initial response highlighted the effectiveness of prompt intervention, the patient’s outcome underscores the lethal potential of compounded critical conditions, particularly when air embolism coexists with severe sepsis. Hyperbaric oxygen therapy was not pursued in this case due to the resolution of the air embolism following catheter removal and positional maneuvers, as well as its limited indication in the setting of sepsis.

Although iatrogenic air embolism is a well-documented complication of CVC, recent advancements suggest that patient-specific factors and procedural dynamics may significantly influence clinical outcomes. Emerging computational fluid dynamics models have provided insights into how microbubbles formed during catheter manipulation interact with blood flow, potentially exacerbating endothelial damage and triggering inflammatory cascades [12]. These findings suggest that even small-volume air embolisms can induce a disproportionate systemic response, particularly in critically ill patients, which may explain why our patient deteriorated despite early intervention. Additionally, newer imaging modalities such as contrast-enhanced US and real-time transesophageal echocardiography have improved the ability to detect and quantify air embolism burden with greater precision than conventional CT scans. This advancement could allow for more targeted interventions, such as catheter-directed aspiration or hyperbaric oxygen therapy, before irreversible organ dysfunction occurs.

Given these insights, preventing air embolism requires a standardized, evidence-based protocol integrating best practices in CVC insertion, maintenance, and removal. Key preventative measures include proper patient positioning (Trendelenburg or supine) to reduce the air entry gradient, the use of US guidance to optimize catheter placement, and closed-system catheter hubs to minimize accidental air entry [13]. A proposed checklist-based protocol, similar to central line-associated bloodstream infection (CLABSI) prevention bundles, should emphasize continuous catheter hub occlusion, breath-hold techniques during catheter removal, and post-procedure monitoring with immediate access to imaging if embolism is suspected. Implementing these strategies in routine clinical practice could reduce iatrogenic air embolism risk and improve patient outcomes, particularly in high-risk ICU settings.

We acknowledge that the air embolism could have been prevented. During insertion, air embolism can be prevented by positioning the patient in the Trendelenburg or supine position, using US guidance, ensuring adequate hydration, minimizing manipulation during and after insertion, and occluding the catheter hub when not in use. Regular inspection of the insertion site is essential to detect any dislodgment or open connections. During CVC removal, the catheter should be withdrawn during expiration or a Valsalva maneuver if the patient is conscious. Immediately after removal, an airtight occlusive dressing should be applied and remain in place for at least 24-72 hours.

## Conclusions

In conclusion, this case highlights a rare but serious complication of CVC insertion, VAE, in a patient presenting with profound shock from concurrent sepsis resulting from UTI. The patient developed air embolism due to a combination of hypovolemia, a landmark-based technique without US guidance, and possible improper catheter hub management. Preventive strategies, including proper patient positioning, adequate volume resuscitation, strict catheter hub occlusion, and the use of US guidance, might have averted this complication. The patient was treated with immediate catheter removal, mechanical ventilation, and positioning maneuvers (Trendelenburg and left lateral decubitus), which partially stabilized her hemodynamics. Hyperbaric oxygen therapy and extracorporeal support were not pursued due to the resolution of the embolism following initial interventions. This case underscores the importance of strict adherence to preventive measures to avoid VAE.
